# Metabolic Conversion of Ceramides in HeLa Cells - A Cholesteryl Phosphocholine Delivery Approach

**DOI:** 10.1371/journal.pone.0143385

**Published:** 2015-11-24

**Authors:** Matti A. Kjellberg, Max Lönnfors, J. Peter Slotte, Peter Mattjus

**Affiliations:** Abo Akademi University, Biochemistry, Faculty of Science and Engineering, Turku, Finland; Medical University of South Carolina, UNITED STATES

## Abstract

Ceramides can be delivered to cultured cells without solvents in the form of complexes with cholesteryl phosphocholine. We have analysed the delivery of three different radiolabeled D-erythro-ceramides (C6-Cer, C10-Cer and C16-Cer) to HeLa cells, and followed their metabolism as well as the cell viability. We found that all three ceramides were successfully taken up by HeLa cells when complexed to CholPC in an equimolar ratio, and show that the ceramides show different rates of cellular uptake and metabolic fate. The C6-Cer had the highest incorporation rate, followed by C10-Cer and C16-Cer, respectively. The subsequent effect on cell viability strongly correlated with the rate of incorporation, where C6-Cer had the strongest apoptotic effects. Low-dose (1 μM) treatment with C6-Cer favoured conversion of the precursor to sphingomyelin, whereas higher concentrations (25–100 μM) yielded increased conversion to C6-glucosylceramide. Similar results were obtained for C10-Cer. In the lower-dose C16-Cer experiments, most of the precursor was degraded, whereas at high-dose concentrations the precursor remained un-metabolized. Using this method, we demonstrate that ceramides with different chain lengths clearly exhibit varying rates of cellular uptake. The cellular fate of the externally delivered ceramides are clearly connected to their rate of incorporation and their subsequent effects on cell viability may be in part determined by their chain length.

## Introduction

Sphingolipids are a major class of lipids that are ubiquitously present in the membranes of eukaryotes, where they function as important structural components and also exhibit important roles as bioactive molecules. Ceramide (Cer) has emerged as a central molecule in the sphingolipid metabolism, by serving as a precursor for all complex sphingolipids and displaying its involvement in a variety of cellular processes, including cell growth, signalling, proliferation, differentiation and apoptosis [1–5]. *De novo* synthesis of ceramide begins at the endoplasmatic reticulum, with a process that yields the long-chain amino-alcohol base, dihydrosphingosine. The dihydrosphingosine base is further modified by the addition of an acyl group by a ceramide synthase, yielding dihydroceramide. Introduction of a trans double bond between carbons 4 and 5 on the amino-alcohol base gives rise to ceramide [6–8]. Degradation of ceramide produces sphingosine, which in turn can be phosphorylated to form sphingosine-1-phosphate, another sphingolipid implicated in cell proliferation and apoptosis [9, 10]. In addition to the *de novo* synthesis pathway, ceramide is produced by the hydrolysis of more complex sphingolipids, especially sphingomyelin (SM) [11].

The involvement of ceramide in the cellular apoptotic processes is evident. Exogenous, apoptotic stimuli have been found to elevate intracellular ceramide levels [12–15] and external delivery of ceramide has been shown to induce apoptosis in cultured cells [2]. On a general level, an increase in intracellular ceramide levels leads to the activation of various protein kinases and phosphatases, which in turn cause caspase cascade activation, dysfunction of organelles and ultimately apoptosis [16]. Ceramide has been implicated in several different cellular functions, and sometimes the implications seem contradictory [17]. This is presumably due to the existence of a multitude of structurally distinct ceramide species. Emerging evidence suggests that different ceramides, in terms of the length of their N-acyl chains, may be destined for different cellular functions in different cell types. In humans, six distinct ceramide synthases (CerS 1 to 6) produce ceramide species of particular chain lengths [18–20]. In addition, the existence of five ceramidases [21], acylsphingosine deacylase, sphingomyelin phosphodiesterase and at least four sphingomyelinases (SMases) [22–24], as well as several other enzymes that use ceramide as a substrate, all add to the complexity of the issue [25, 26]. The task of elucidating the precise roles of the many differing ceramides is therefore particularly challenging.

Ceramide is a highly hydrophobic molecule and exhibits very poor solubility in water. As a result, studies done on ceramide bioactivity have been methodologically limited, due to the difficulty of exogenous delivery to cultured cells. The role of endogenous ceramide has been investigated through the manipulation of various enzymes involved in ceramide metabolism, such as the endogenous SMases [27, 28], or with the help of purified, bacterial SMases [29, 30]. In addition, the external delivery of the more water-soluble and non-physiological short-chain C2-Cer and C6-Cer to cells, is achievable with the help of organic solvents [31, 32].

C6-Cer loading of human A549 adenocarcinoma epithelial cells generate higher endogenous ceramide (C16:0 and C24:1) levels due to biochemical recycling of the sphingosine backbone into long-chain ceramides, and not due to elongation of the short acyl acid chain [33]. It is still not known which of the five different human ceramidases that are involved in this process, the acidic or neutral or the three alkaline. Neutral ceramidase activity has been found at the outer leaflet of the plasma membrane, however it appears not to act on short ceramides [34]. The acidic form is localized to the lysosomes, and the three alkaline in the ER-Golgi compartments. The alkaline isoforms favour long or very long ceramides [35, 36]. It is likely that the remodelling of short-chain ceramides takes place in the lysosomes by the acidic ceramidase, the dysfunctional enzyme in Farber disease [37].

Lönnfors et al. previously demonstrated that C6-Cer and C16-Cer can form stable bilayers with cholesteryl phosphocholine (CholPC) in a 1:1 molar ratio [38]. Ceramide and cholesterol interact unfavourably in the absence of large polar headgroup phospholipids (such as SM). The phosphocholine headgroup on the CholPC molecule acts to protect the ceramide from the aqueous environment and allows for the formation of bilayers (umbrella effect) [39]. Similarly, CholPC can form bilayers together with dimyristoglycerol and free cholesterol [40]. In another study, Sukumaran and co-workers demonstrated that complexes formed by short-chain C6-Cer and CholPC can be used to deliver ceramide to cells in culture [41]. The observed inhibition of cell growth and the apoptotic effects were significantly more drastic in the Cer/CholPC treated cells when compared to C6-Cer that was solvent-dissolved. This data suggest that complexing C6-Cer with CholPC offers a higher bioavailability of ceramide to cells, when compared to C6-Cer/DMSO formulations.

Here we have analysed the uptake of radiolabeled C6-Cer, C10-Cer and C16-Cer in complex with CholPC by HeLa cells. In addition, we analysed the cellular metabolism of the incorporated ceramides and assessed how the different Cer/CholPC formulations affect cell viability. We found that long-chain ceramides can be introduced to cells when complexed to CholPC. We demonstrate that ceramides with different chain lengths exhibit different rates of cellular uptake. The cellular fate of the delivered ceramides, and their subsequent effects on cell viability, strongly correlates with their rate of incorporation.

## Materials and Methods

### Material

All chemical reagents were of analytical grade or higher. Lipid standards where from Avanti Polar Lipids (Alabaster, USA) or Matreya LLC (Pleasant Gap, USA). Cholesteryl phosphocholine (CholPC) was synthesized as described by Lönnfors et al. [38], or obtained from Avanti Polar Lipids. D-erythro-sphingosine and hexanoic acid were purchased from Larodan (Malmö, Sweden). [3-^3^H]D-erythro-sphingosine (15–30 Ci/mmol) and [9–10,^3^H]hexadecanoic acid (30–60 Ci/mmol) were obtained from PerkinElmer (Waltham, MA, USA). Decanoic and hexadecanoic acids were obtained from Sigma-Aldrich (St. Louis, MO, USA). Organic solvents were from Rathburn Chemicals Ltd (Walkerburn Scotland). HeLa cells (ATCC CCL-2, LGC Standards) were cultured in Dulbecco’s Modified Eagle’s medium (DMEM, Sigma-Aldrich) supplemented with penicillin (50 U/ml), streptomycin (50 U/ml), 4 mM L-glutamine and 10% fetal calf serum (FCS, all from Sigma-Aldrich) prior to the experiments. Chloroquine and ceranib-2 were purchased from Sigma-Aldrich and fumonisin B1 was purchased from Enzo Life Sciences (Farmingdale, NY, USA).

### Synthesis of [^3^H]-labeled ceramides

C6-[^3^H]Cer, C10-[^3^H]Cer, and C16-[^3^H]Cer were prepared from [3-^3^H]D-erythro-sphingosine and hexanoic, decanoic and hexadecanoic acids, respectively, using *N*,*N'*-dicyclohexylcarbodiimide and triethylamine as catalysts [42]. [^3^H]palmitoyl ceramide (C16-Cer) was prepared from sphingosine and [9–10,^3^H]hexadecanoic acid, using *N*,*N'*-dicyclohexylcarbodiimide and triethylamine as catalysts [42]. The products were purified by preparative HPLC on a C18 phase, using methanol as solvent. Purity was assessed by analytical HPLC, and molecular identity by ESI-MS.

### Preparation of ceramide-cholesterol phosphocholine bilayers

CholPC and ceramide were kept in hexane-isopropanol (3:2 by volume) solutions and stored at -20°C until used. Cer/CholPC complexes of desired concentration were prepared from the stock solutions. The appropriate amount of each lipid was dried under nitrogen in a glass tube, redissolved in chloroform to ensure proper lipid mixing and dried again. The dehydrated lipid film was then hydrated in PBS, pH 7.4, at 55°C for 20 minutes and then sonicated for 5 minutes in a FinnSonic M3 bath sonicator (FinnSonic Oy, Lahti, Finland) at the same temperature. The solution was further sonicated for 10 minutes using a Branson 250 probe sonifier at room temperature (Emerson Industrial Automation, St. Louis, MO, USA). The resulting clear solution was then immediately centrifuged at 12 000 rpm for 10 minutes using a table top microfuge, to remove titanium probe particles and any undispersed lipids. The solution was transferred to a glass tube and kept at RT and used within 1 h. Prior to use, the ceramide concentration in the solution was re-calculated based on the specific activity of the radiolabeled ceramide.

### Labeling of cells, lipid and protein extraction

HeLa cells were cultured until 90% confluence before the start of each treatment. The previously prepared Cer/CholPC stock solution was added to DMEM supplemented with L-glutamine, antibiotics and 10% FCS, to achieve the desired concentration of ceramide. The ceramide concentration in the growth medium was verified by measuring the total radioactivity in solution. HeLa cells were washed twice in PBS, pH 7.4, and then exposed to the growth medium containing the Cer/CholPC complex. After treatment, the cells were washed twice in PBS and the cell dishes were dried to completion in a fume hood. The total lipids were extracted directly from the cell dishes using hexane-isopropanol (3:2 by volume). The extracted lipids were transferred to glass tubes and dried under a stream of nitrogen. The dried lipids were stored at -20°C until analysed. After lipid extraction, the cellular proteins were extracted with 0.1 M NaOH and the protein content was analysed by the Lowry method [43]. All cellular treatments were done in the presence of 10% FCS.

### Identification and quantification of lipid species with HPTLC

The dried lipids were redissolved in hexane-isopropanol (3:2 by volume) and analysed on HPTLC plates using a solvent system consisting of chloroform:methanol:acetone:acetic acid:water (10:2:4:2:1). The different lipids were identified by standards, run in parallel with the samples. Glycosphingolipid (GSL) migration was visualized using orcinol spray (0.2% orcinol in a 20% H_2_SO_4_ solution) and heating the plate on 120°C for 5 minutes. For the other lipids, subsequent iodine or cupric-acid staining was used for visualization. The marked silica spots were scraped into Optiphase ‘Hi phase’ scintillation fluid (PerkinElmer-Wallac, Turku, Finland) and the radioactivity was measured using a liquid scintillation counter (PerkinElmer-Wallac, Turku, Finland). The counts per minute (cpm) obtained were either normalized to the total protein content for each respective sample, or presented as a percentage of the total signal per sample.

### Cell viability assay

A resazurin reduction assay (alarmaBlue, Life Technologies) was used to investigate cytotoxicity of C6-Cer, C10-Cer, and C16-Cer towards HeLa cells. The assay is based on the reduction of resazurin to the fluorescent molecule resorufin by viable cells. Nonviable cells lose their metabolic capability to reduce resazurin and no fluorescent resorufin is formed. HeLa cells were seeded to 50000 cells per well in a 24-well plate and were allowed to adhere and grow for 24h to a confluence of about 50%. The cells were treated for 22 hours with the different ceramides, cholesterol or PBS-vehicle. After the 22-hour incubation, the reazurin conversion assay was performed according to the manufacturer's instruction. The fluorescence was measured by a Varioskan Flash Multimode Reader (Thermo Scientific) using an excitation wavelength of 560 nm and an emission wavelength of 590 nm. Each experiment was performed at least 3 times, with 4 replicates each.

### Dot blot analysis of C6- and C10-GlcCer

High-dose C6- and C10-GlcCer (50 μM and 100 μM, respectively) treated samples were analysed by HPTLC as described above. The HPTLC plate was stained by iodine vapour, and the lipid spots corresponding C6- and C10-GlcCer were scraped into glass tubes. The lipids were extracted from the scraped silica using chloroform-methanol (2:1 by volume) and dried under a nitrogen stream. The dried lipids were redissolved in hexane-isopropanol (3:2 by volume) and appropriate amounts were dotted onto a nitrocellulose membrane, alongside GlcCer and GalCer standards (2 nmol/dot). The lipids were visualized by immunoblotting, using commercial rabbit anti-GlcCer and rabbit anti-GalCer antibodies (Glycobiotech GmbH), a HRP-conjugated anti-rabbit secondary antibody (Thermo Scientific) and enhanced chemiluminescence detection (SuperSignal West Femto Maximum Sensitivity Substrate, Thermo Scientific).

### Statistical Analysis

Conventional statistical analysis was performed to calculate mean values and standard errors of the mean. Differences between data points were tested for significance using Student’s t-test for unpaired observations. Results are presented as means of at least three individual measurements with SEM.

## Results

### Cellular uptake and fate of [^3^H]C6-Cer, [^3^H]C10-Cer and [^3^H]C16-Cer

**Fig 1:**
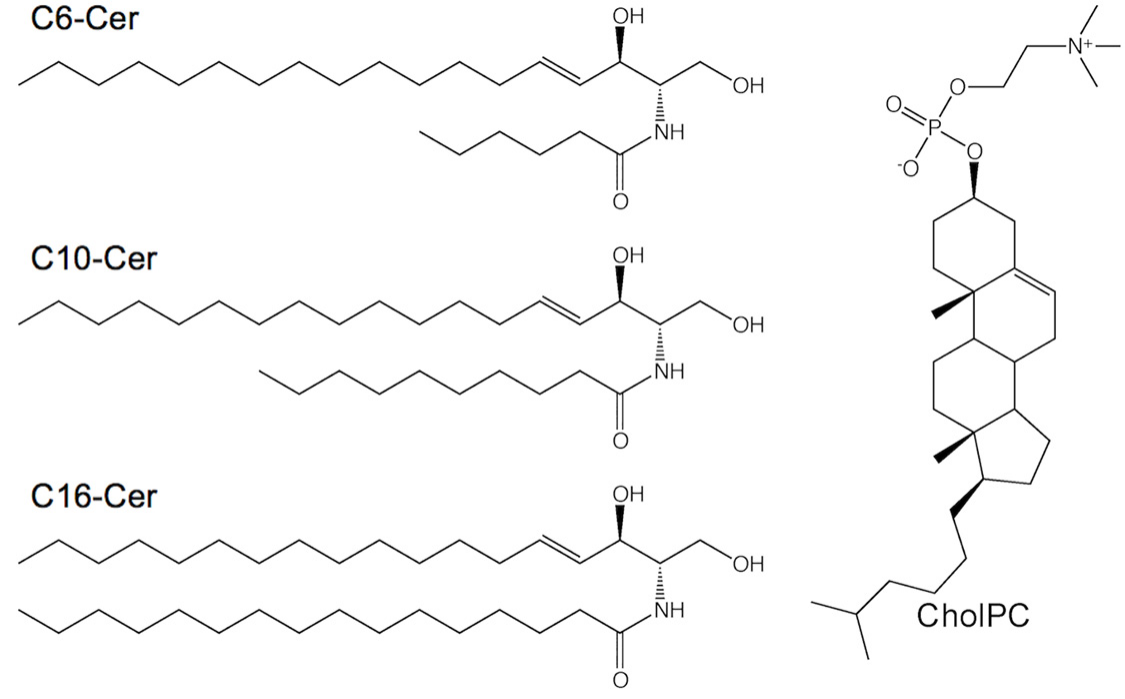
Ceramides with different amide-linked acyl chains and cholesteryl phosphocholine used in this study.

Previously, Sukumaran and co-workers analysed whether C6-Cer could be introduced into cells in a solvent-free manner, by complexation with CholPC in vesicles [41]. They showed that C6-Cer in complex with CholPC resulted in a more dramatic apoptotic effect, compared to C6-Cer that was dissolved in DMSO and introduced by dispersion into the growth medium, demonstrating that the Cer/CholPC formulation appears to be superior to previous solvent delivery approaches. Here, we expand upon the methodology, and analyse the incorporation of longer-chain ceramides (C10-Cer and C16-Cer) into HeLa cells, using the same complexation approach (**Fig 1**).

**Fig 2:**
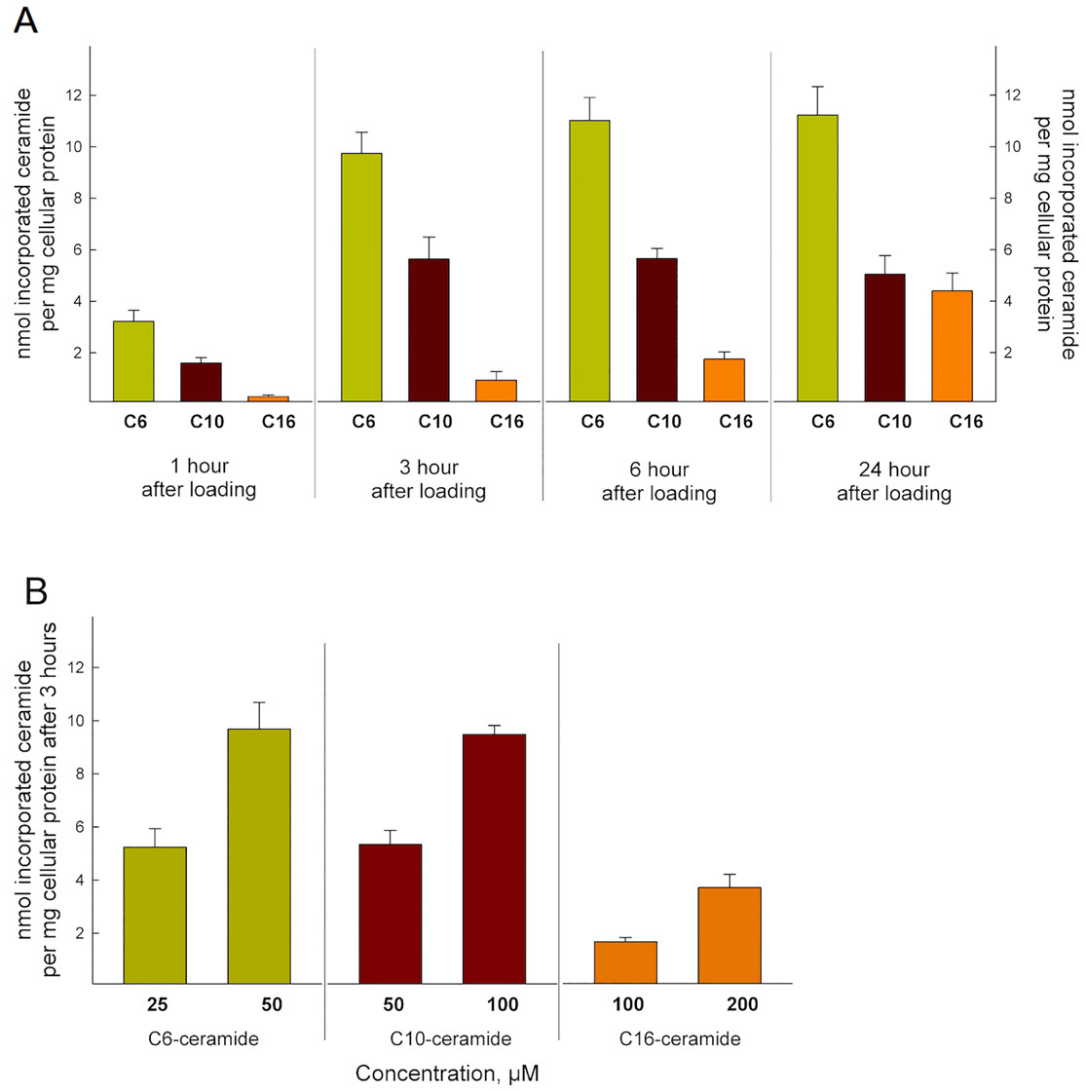
Ceramide precursor uptake. **(A)** The amounts of radiolabeled C6-, C10- and C16-Cer uptake as nmol ceramide per mg total protein as a function of time. HeLa cells were labeled with [^3^H]Cer (50 μM), with the radionuclide in positions 4 and 5 of the sphingosine backbone. The uptake of ceramide by HeLa cells was analysed by HPTLC and scintillation counting. **(B)** Amount of ceramide precursor incorporation into HeLa cells at different loading concentrations of ceramides after a 3-hour incubation. The data for the incorporation of the radiolabeled [^3^H]Cers are from at least three different experiments.

To measure the cellular uptake of the different ceramides, HeLa cells were subjected to 50 μM of the various chain length, [^3^H]sphingosine labeled ceramides, complexed with CholPC in a 1:1 molar ratio. The total radiolabel uptake was analysed after 1, 3, 6 and 24 hours (**Fig 2A**). [^3^H]C6-Cer uptake was the most rapid of the three ceramides, reaching a plateau within 6 hours from the start of the experiment, **Fig 2A**. The rate of incorporation for [^3^H]C10-Cer and [^3^H]C16-Cer at 1 and 3 h were roughly 50% and 5%, respectively, when compared to [^3^H]C6-Cer. Interestingly, both [^3^H]C10-Cer and [^3^H]C16-Cer uptake seemed to plateau at around 50% of the maximum incorporation rate of [^3^H]C6-Cer. It is worth noting that cell detachment at 24 hours in the [^3^H]C6-Cer samples was significantly higher when compared to the [^3^H]C10-Cer and [^3^H]C16-Cer treated cells (data not shown). We also analysed the amount of ceramide precursor incorporation into HeLa cells at two different concentrations of ceramides after a 3-hour incubation (**Fig 2B**). The cells did not take up the longer [^3^H]C16-Cer to the same extent as [^3^H]C6-Cer, or [^3^H]C10-Cer, not even at higher concentrations.

**Fig 3:**
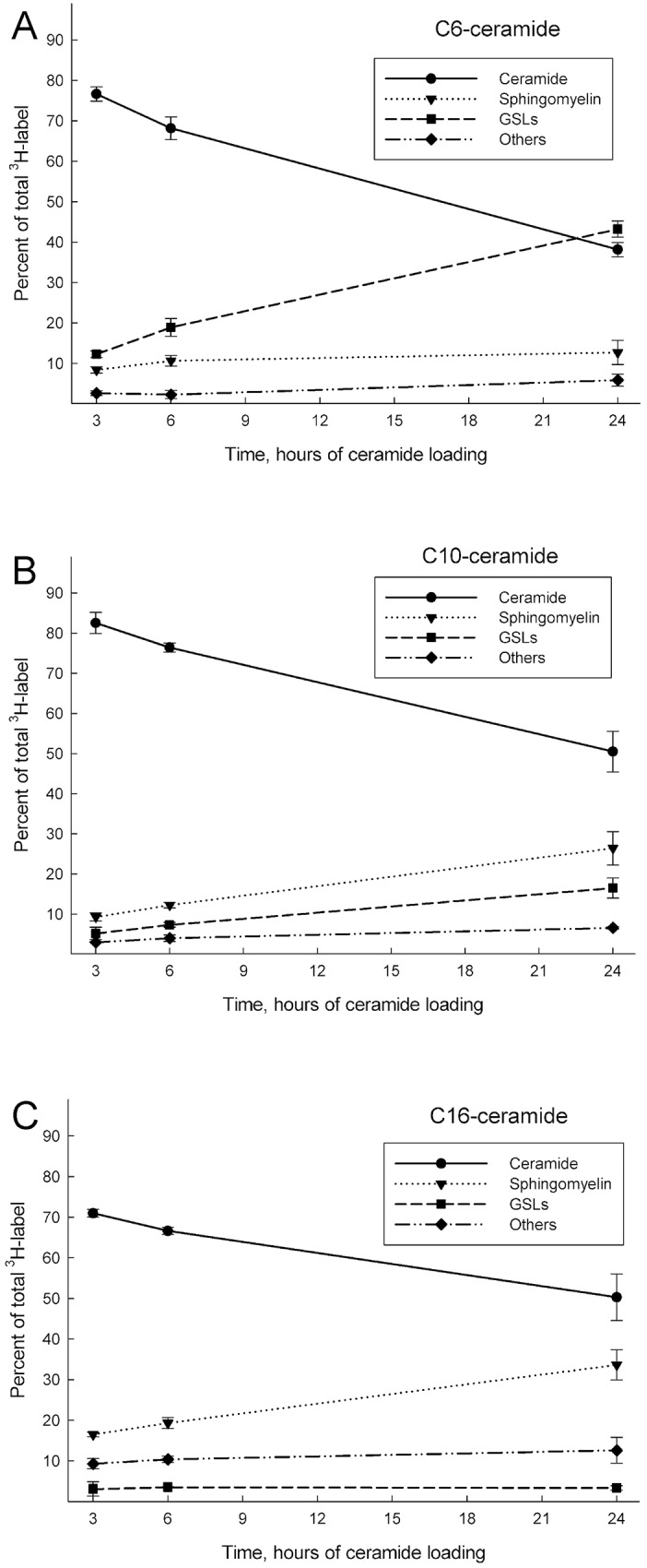
Radiolabeled ceramides with different acyl chain lengths metabolize into sphingolipids as a function of time. HeLa cells were loaded with C6-Cer **(A)**, C10-Cer **(B)** and C16-Cer **(C)** in CholPC complexes. The [^3^H]sphingosine labeled ceramides (50 μM) were allowed to be taken up by HeLa cells for 3, 6 and 24 hours, and the incorporation was followed by HPTLC analysis. The amount of each lipid is presented as a percentage of the total radioactivity in the sample. GSLs = GlcCer, GalCer, GlcCer-OH and GalCer-OH. Other = the remaining lane. The data is from at least three different experiments.

Next we examined whether ceramides that are introduced to HeLa cells are metabolized to other sphingolipid classes. Cells were treated as described above (50 μM, 3, 6 and 24 h), whereafter total lipids were extracted and analysed using HPTLC and scintillation counting. **S1 Fig** shows a representative HPTLC plate visualized using orcinol spray and subsequent cupric acid staining. The boxed regions correspond to the different lipids that were scraped off the plate (dashed lines represent “Other”). **Fig 3**shows the quantified radioactivity in the different lipid species analysed, where each lipid is presented as a percentage of the total radioactivity in the sample (total in lane). As can be seen, the incorporated ceramide is metabolized and the radioactive label is distributed into other sphingolipids as a function of time. In the case of [^3^H]C6-Cer (**Fig 3A**), at 24 hours, over 40% of the radioactive signal could be found in the simple GSLs, i.e. glucosylceramide (GlcCer) and galactosylceramide (GalCer). However, for [^3^H]C10-Cer (**Fig 3B**) and [^3^H]C16-Cer (**Fig 3C**), the conversion favoured SM over simple GSLs.

### Metabolic fate of exogenous ceramide is affected by the rate of incorporation

From the results presented in **Fig 2**, it is evident that the rate of incorporation was different between the varying ceramides. The short [^3^H]C6-Cer had the most rapid uptake and was the only precursor which resulted in a large portion of the signal to be found in the simple GSLs after a 24 hour treatment (**Fig 3A**). Cancerous cells in culture have been shown to rapidly convert C6-Cer to either C6-SM or C6-GlcCer [44]. In some cancer cells the conversion route is determined by the initial C6-Cer concentration in the culture medium, where lower concentrations facilitate conversion to SM and higher concentrations result in conversion to GlcCer [44]. We therefore decided to analyse whether treating HeLa cells with different concentrations of [^3^H]C6-Cer, [^3^H]C10-Cer and [^3^H]C16-Cer would yield similar metabolic effects.

**Fig 4:**
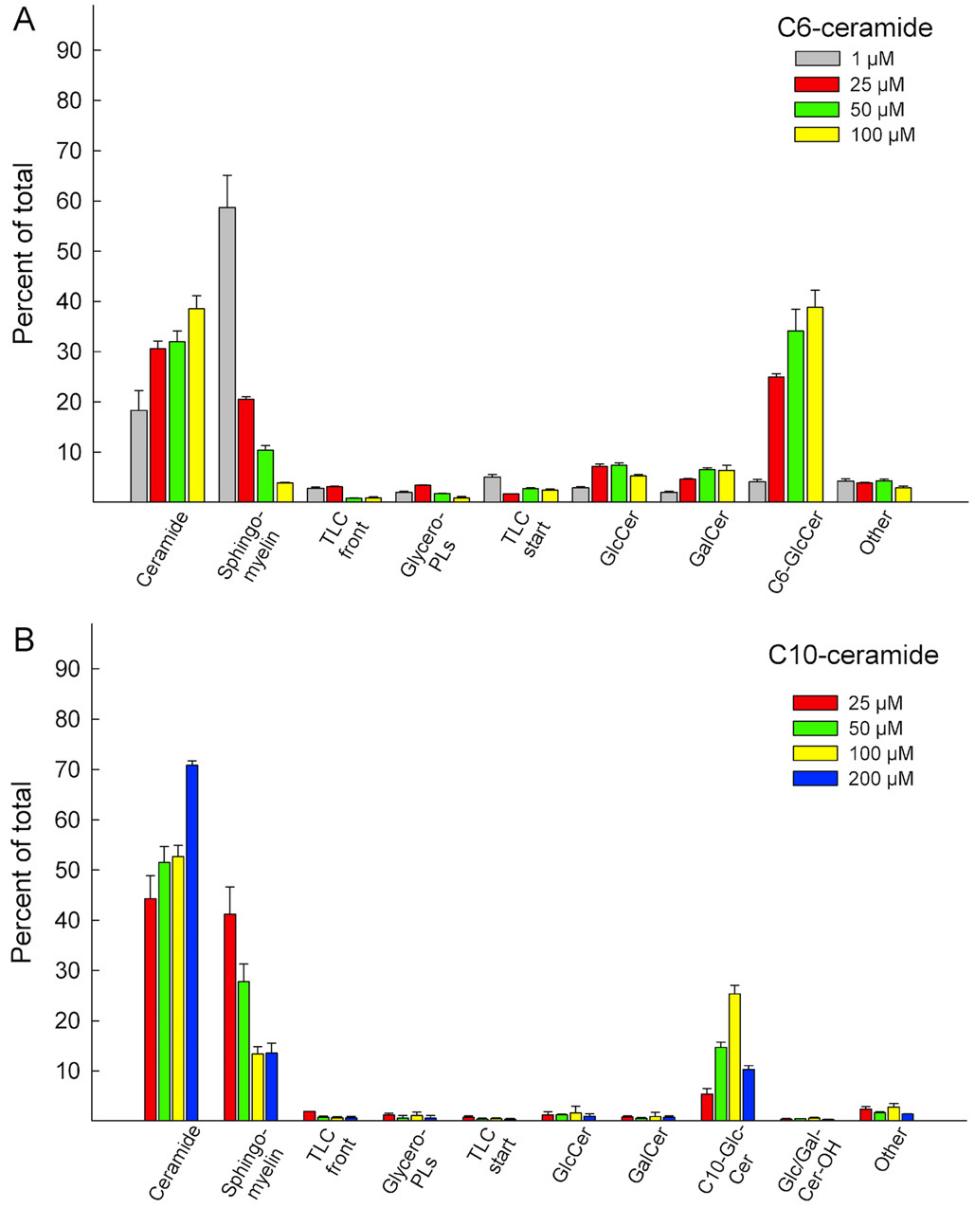
HeLa cells C6- and C10-Cer precursor uptake. Analysis of **(A)** C6-Cer and **(B)** C10-Cer incorporation at different concentrations. The ceramides were [^3^H]-labeled in the sphingosine part. After 24 h of ceramide uptake the total lipids were extracted and analysed by HPTLC. “Start” and “Front” refer to the labeled lipids remaining on the application spot, or that were eluted along with the solvent front, “Other” refers to the traces of radiolabeled lipids between the identified spots (see **S1 Fig**). The data is from at least three different experiments.

**Fig 5:**
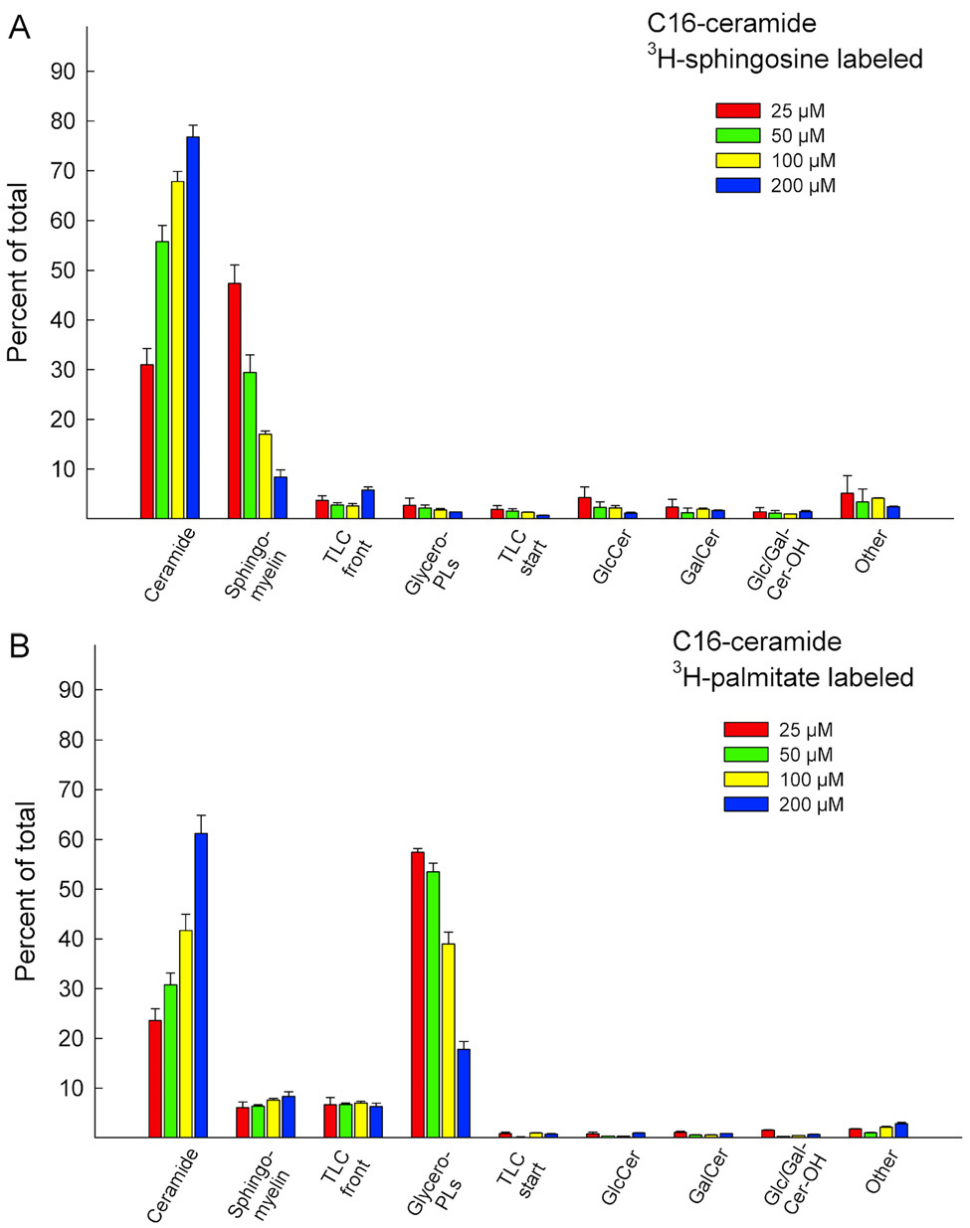
Analysis of C16-Cer precursor incorporation at different concentrations. The ceramide was either **(A)** [^3^H]-labeled in the sphingosine backbone or in the **(B)** palmitic acid portion. After 24 h of ceramide uptake the total lipids were extracted and analysed by HPTLC. “Start” and “Front” refer to the labeled lipids remaining on the application spot, or that were eluted along the solvent front, “Other” refers to the traces of radiolabeled lipids between the identified spots (see **S1 Fig**). The data is from at least three different experiments.

HeLa cells were treated with varying concentrations of the respective ceramides for 24 hours, as indicated in **Figs 4 and 5**. For [^3^H]C6-Cer and [^3^H]C10-Cer, the radiolabel resided on the sphingosine base (**Fig 4A and 4B**). For [^3^H]C16-Cer, both a sphingosine and a palmitic acid labeled ceramide was used, in separate experiments (**Fig 5A and 5B)**. Suitable ceramide concentrations were estimated based on the incorporation rates in **Fig 2**, and from previous works in the literature [44]. As expected, a low-dose treatment with [^3^H]C6-Cer favoured conversion to SM (**Fig 4A, grey bars**), whereas a higher concentrations yielded increasing signals in C6-GlcCer (**Fig 4A, red, green and yellow bars**). Similar results were obtained for [^3^H]C10-Cer (**Fig 4B**). Interestingly, a higher-dose treatment with [^3^H]C16-Cer, did not result in a marked increase of signal to be found in the simple GSLs (**Fig 5A**). Instead, the majority of the signal remained in ceramide. This was evident for both the sphingosine (**Fig 5A**) and the palmitic acid (**Fig 5B**) labeled [^3^H]C16-ceramide. In the [^3^H]C16-Cer treated cells, where the radiolabel resided on the acyl chain of the ceramide, a significant portion of the radioactivity could be found in the glycerophospholipids (**Fig 5B**). This suggests that [^3^H]C16-Cer is subject to significant degradation when delivered from Cer/CholPC complexes. This issue was further addressed by using the ceramidase inhibitor ceranib-2 and the lysosome inhibitor chloroquine. We found that when HeLa cells were treated with ceranib-2 (20 μM) or chloroquine (20 μM) and subsequently loaded with [^3^H]C16-Cer ([^3^H]palmitic acid labeled [^3^H-PA], 50 μM, 8 h), a decrease in the ceramide degradation occurred (higher content of ceramide remained in the cells, black bars **S2 Fig**). In the chloroquine treated cells, in addition to an increase in the ceramide content, the metabolic conversion of [^3^H]C16-Cer shifted towards that of SM (right graph, red bars, **S2 Fig**). In the ceranib-2 treated cells, no shift in SM content was observable, but instead an overall increase in ceramide was seen (middle graph, **S2 Fig**). When the ceramide degradation in the lysosomes was inhibited by ceranib-2 or chloroquine, a much lower incorporation of [^3^H]palmitic acid could be seen in glycerophospholipids as well (green bars, **S2 Fig**). Additionally, fumonisin B1 (FB1) was used to test whether the inhibition of ceramide synthesis would subsequently inhibit the putatively liberated [^3^H]sphingosine from being re-introduced into ceramide and SM (**3**). **S3A Fig** shows that FB1 treatment (200 μM FB1, 50 μM [^3^H]C16-Cer, 24 h) decreased radioactivity in SM by roughly 50%, when the radionuclide resided on the sphingosine base. When the radionuclide resided in the palmitic acid, FB1 treatment did not yield drastic changes in the signal distribution, however, radioactivity in the glycerophospholipids did increase slightly (**S3B Fig**). The inhibitory effectivity of FB1 at 200 μM was verified by analysing [^3^H]sphingosine incorporation into HeLa cells, in the presence and absence of FB1 (data not shown).

In the higher-dose experiments, both C6-GlcCer and C10-GlcCer were clearly distinguishable from the longer-chain GlcCer- and GalCer-standards on the HPTLC plate, as indicated by orcinol staining (**S4A Fig**). The C6-GlcCer and C10-GlcCer lipid spots were experimentally validated by immunodetection using rabbit anti-GlcCer and rabbit anti-GalCer antibodies (**S4B Fig**).

### Cell viability in response to increasing concentration of C6-, C10- and C16-Cer/CholPC formulations

As was previously reported, complexation of C6-Cer with CholPC gave rise to higher apoptotic effects and more drastic inhibition of cell growth in HeLa and FRTL-5 cells, when compared to DMSO-solubilised C6-Cer treatment [41]. Lower-dose treatments using the various ceramides did not result in noticeable cell detachment or morphological changes (visual observation, data not shown), whereas respective high-dose treatments did. We therefore investigated what effects the treatments had on cell viability, using a commercial reazurin-conversion assay.

**Fig 6:**
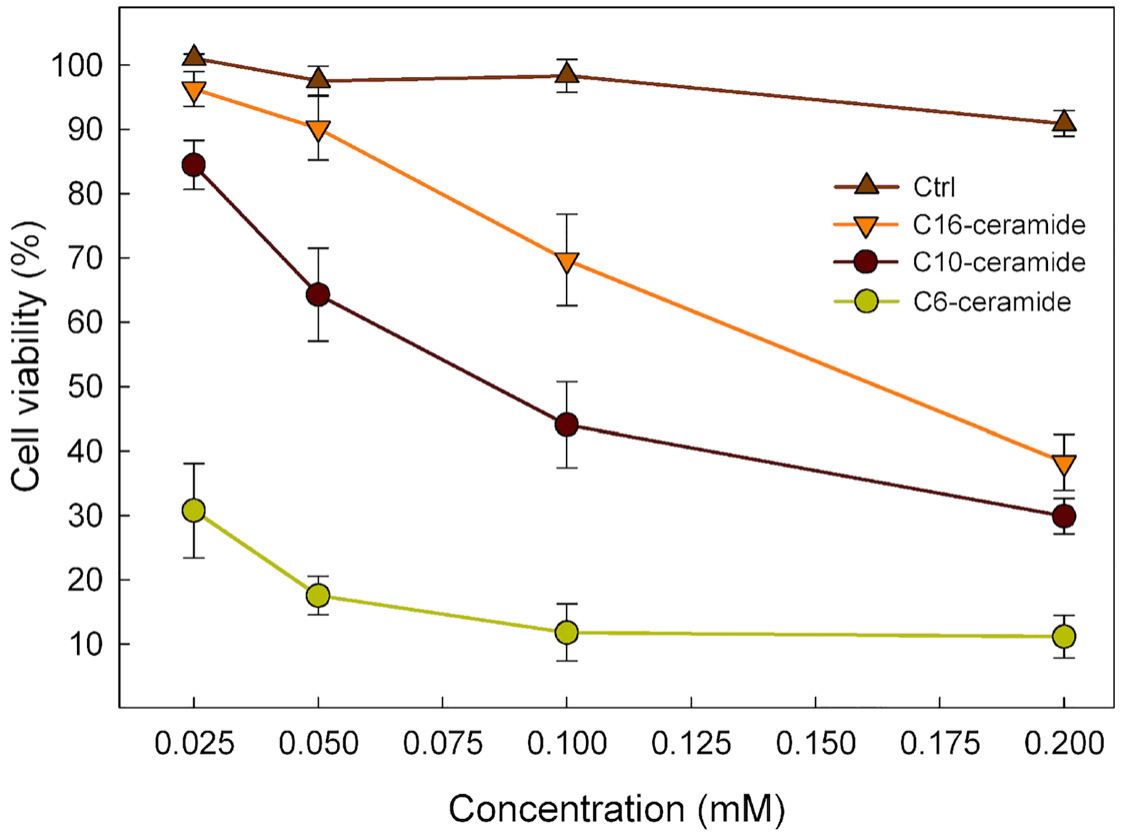
Cytotoxic effect of ceramides. HeLa cells were treated with increasing concentrations of C6-, C10-, C16-Cer or cholesterol, complexed with CholPC, for 22 h and compared to vehicle (PBS) treated cells. Subsequently, a resazurin reduction assay was performed. Viability of the HeLa cells is represented by mean ± SEM of at least 3 independent experiments and is expressed as percentage survival of PBS control.

Cells were treated with varying doses of cholesterol (as a control), C6-Cer, C10-Cer or C16-Cer (25–200 μM) in complex with CholPC (**Fig 6**). The cell viability of each sample was normalized to respective vehicle (PBS) treated controls. The treatments were carried out for 22 hours, where after cell viability was assayed. The results show that all ceramide treatments had a dose-dependent effect on cell viability, where the C6-Cer/CholPC formulation was the most effective at decreasing viability. The cholesterol/CholPC vesicles, even at higher doses (200 μM), did not significantly affect cell proliferation. As shown previously, the incorporation speed of the three different ceramide species varied significantly (**Fig 2**). C16-Cer, despite exhibiting an incorporation rate of only 5% when compared to C6-Cer, was still able to induce a marked reduction in cell viability at a concentration of 200 μM

## Discussion

Ceramides are important bioactive molecules that are implicated in many cellular processes, including cell growth, senescence, necrosis, differentiation, proliferation and apoptosis [1–5]. The study of ceramides and their function has been hampered by the molecule's inherent physical attributes. Ceramide is highly hydrophobic and therefore does not efficiently transfer to cells when dispersed in aqueous growth medium. Instead, non-physiological short-chain ceramides have been used extensively to study the effects of ceramide in cells, due to their water-soluble nature. Cyclodextrins have been utilized for both the loading and the depletion of cholesterol in cellular studies [45, 46]. However, ceramides do not seem to favour the formation of water-soluble complexes with cyclodextrins, and therefore cyclodextrins offer only a limited solution for ceramide delivery [47]. Liposomal complexes of ceramides have been previously used to deliver C16-Cer into cells [48]. While high ceramide content liposomes (up to 50 mol% ceramide) were successfully produced, cells with enhanced liposome endocytosis had to be utilized for efficient uptake and maximal cellular response.

We have previously shown that C6-Cer and C16-Cer can form apparently stable, fluid bilayers with CholPC in a 1:1 molar ratio [38]. We previously also demonstrated that C6-Cer can be introduced to cells efficiently when complexed with CholPC and that the C6-Cer/CholPC formulation was superior to the C6-Cer/DMSO formulation in all cellular responses studied [41]. In the present study, we demonstrate that the same methodology of complexation can be utilized for introducing longer-chain C10-Cer and C16-Cer into cells. In addition, we show that the rate of the uptake seems to be a defining factor in how the cells metabolize the introduced ceramide.

Our results indicate that all three ceramides could successfully be delivered to cells by virtue of their CholPC formulations. The short-chain C6-Cer had the highest incorporation rate, followed by the C10-Cer and the C16-Cer, respectively. Sukumaran and co-workers previously suggested that the method of C6-ceramide uptake mainly takes place through monomeric exchange between the ceramide/CholPC bilayer and the plasma membrane [41]. This suggestion is supported by the results presented in this study. The acyl chain length of lipids correlate with their transfer efficiency between membrane structures [49, 50]. Longer-chain ceramides should therefore transfer between membrane structures more slowly than shorter-chain ones. However, fusion of Cer/CholPC vesicles with the plasma membrane, and subsequent internalization, cannot be completely ruled out as an uptake method, since it was previously reported that CholPC also was internalized by the cells [41].

The metabolism of the externally delivered ceramide was not studied previously [41]. We therefore decided to analyse and compare the cellular fates of the different ceramides. At concentrations of 50 μM, there were marked differences in how the different ceramides ([^3^H]sphingosine labeled), as a function of time, were metabolized by the cells. At the 24 hour mark, in the samples that had been treated with [^3^H]C6-Cer, more than 40% of the radioactive signal could be found in the simple GSL (Glc/GalCer & Glc/GalCer-OH) spots. In the comparable [^3^H]C10-Cer and the [^3^H]C16-Cer treated samples, SM has the strongest radiolabel incorporation of the analysed lipids.

In some cancer cells, C6-Cer metabolism has been shown to be subject to the initial C6-Cer concentrations the cells are exposed to [44]. Lower concentrations seem to facilitate conversion of C6-Cer to C6-SM, whereas higher concentrations favour conversion to C6-GlcCer. Since the longer-chain [^3^H]C10-Cer and [^3^H]C16-Cers had markedly lower incorporation rates, when compared to [^3^H]C6-Cer, we decided to analyse whether increased concentrations of [^3^H]C10-Cer and [^3^H]C16-Cer would cause a change in how the ceramides were metabolized. In addition, a more in-depth analysis of the precursor distribution was performed. The results for [^3^H]C6-Cer were in accordance with previously published data, where a lower-dose treatment (1 μM) yielded the largest incorporation into SM and higher doses (25–100 μM) yielded high incorporation in C6-GlcCer. At concentrations exceeding 100 μM [^3^H]C6-Cer, analysis was hindered due to nearly complete cell detachment and death. The [^3^H]C10-Cer treated samples gave similar results. At 200 μM [^3^H]C10-Cer, however, the precursor distribution shifted from C10-GlcCer towards ceramide. For the [^3^H]C16-Cer experiments, in addition to using a [^3^H]sphingosine labeled ceramide, a [^3^H]palmitic acid labeled ceramide was used. For the 25–50 μM [^3^H]C16-Cer experiments, most of the signal was found in either SM ([^3^H]sphingosine labeled ceramide) or the glycerophospholipids ([^3^H]palmitic acid labeled ceramide). As concentrations were increased, the precursor remained un-metabolized. The high incorporation of precursor in the glycerophospholipids in the lower-dose [^3^H]palmitic acid labeled C16-Cer samples strongly suggests that [^3^H]C16-Cer was subjected to degradation. This is supported by results form experiments where ceramide degradation was inhibited using the ceramidase inhibitor ceranib-2 and the lysosomal inhibitor chloroquine. Additionally, the ceramide synthase inhibitor FB1 was able to decrease SM radioactivity in cells treated with [^3^H]sphingosine labeled C16-Cer, whereas when [^3^H]palmitic acid labeled C16-Cer was used, no significant changes were observed. Seemingly, [^3^H]-sphingosine labeled C16-Cer is degraded in a similar fashion to that of [^3^H]palmitic acid labeled C16-Cer. The radioactivity observed in SM in these experiments most likely comes from recycled [^3^H]sphingosine, at least to a significant degree. Whether the same phenomenon can be observed in the lower-dose [^3^H]C6-Cer and [^3^H]C10-Cer samples is not discernible from these experiments.

In the [^3^H]C6-Cer and [^3^H]C10-Cer higher-dose samples, both C6-GlcCer and C10-GlcCer, respectively, were clearly observable on the HPTLC plates (iodine or orcinol staining). In addition, they were also clearly distinguishable from the long-chain GlcCer and GalCer standards that were run in parallel with the samples. Therefore, we assume that the radioactivity found in C6-GlcCer and C10-GlcCer accurately represents [^3^H]C6-Cer and [^3^H]C10-Cer that has been directly glycosylated to GlcCer. Interestingly, for both [^3^H]C6-Cer and [^3^H]C10-Cer, higher-dose treated samples showed a relatively high signal in the endogenous simple GLSs. This would suggest that even when exposed to high concentrations of [^3^H]C6-Cer or [^3^H]C10-Cer, some degradation of the ceramide, and subsequent recycling of the [^3^H]sphingosine base occurs. It is interesting to note that a high-dose treatment with [^3^H]C6-Cer or [^3^H]C10-Cer results in an accumulation of precursor in C6-GlcCer and C10-GlcCer, respectively. A significantly weaker signal could be found in the more complex GSLs (“Start” spot on the HPTLC plate) and in lactosylceramide (“Other” spot), to which GlcCer is an immediate precursor. This suggests that, at least under these conditions, C6-GlcCer and C10-GlcCer are not used as precursors for higher GSLs. This clearly warrants further investigations.

The anti-proliferative and pro-apoptotic effects that ceramides have on cultured cells are evident. Apoptotic stimuli have been found to increase the levels of intracellular ceramide [12–14] and exogenous treatment with short-chain ceramides cause cells to undergo apoptosis [2]. Ceramide takes part in signalling events, both in the extrinsic and intrinsic apoptotic pathways [12, 16, 51]. The methods of action include inhibition of survival pathways, such as pAkt inhibition [52, 53], and permabilization of the mitochondrial outer membranes. Ceramide has the ability to form pores in membrane bilayers, which in the case of the mitochondria results in disruption of its function and release of apoptogenic factors [54–57]. Previously, a dramatic increase in the anti-proliferative and proapoptotic effects in HeLa and FRTL-5 cells was observed, when exposed to C6-Cer/CholPC formulations [41]. In the present study we have investigated what effects C10-Cer and C16-Cer/CholPC formulations have on the viability of HeLa cells, and compared them to C6-Cer. From our results, it is evident that the rate of ceramide incorporation is a major determinant for its effect on cell viability. C6-Cer has the fastest incorporation rate and exhibits the strongest anti-proliferative effects, followed by C10-Cer and C16-Cer, respectively. It would also seem that C6-Cer intrinsically has stronger anti-proliferative effects on HeLa cells, when compared to C10-Cer. This is evident when comparing the effects the two ceramides has on cell viability, when the rate of incorporation (based on total uptake after 3 h) has been accounted for. C6-Cer has a stronger effect on HeLa cell viability, even when C10-Cer has a higher incorporation level (25 μM C6-Cer versus 100 μM C10-Cer). It is also interesting to note that the effects that a 200 μM C16-Cer treatment had on the cell viability were comparable to that of a 100 μM of C10-Cer, even when C10-Cer uptake was higher than that of C16-Cer. One explanation for the observed phenomenon may lie in how the metabolism of C16-Cer (or rather, the lack thereof) in HeLa cells occurs. In high-dose treatments with [^3^H]C16-Cer (200 μM), most of the signal remained as ceramide after 24 hours. In the comparable C6-Cer and C10-Cer experiments, the cells converted the ceramide to GlcCer. Glycosylation of ceramide may be a method by which the cell is able to suppress the anti-proliferative effects of high doses of externally introduced ceramides. Both GlcCer-synthase inhibitors, as well as P-gp antagonists, have been shown to enhance the ceramide-induced apoptotic effects when co-administered with short-chain ceramides [44, 58, 59]. Presumably, when the synthesis of GlcCer is blocked, the higher accumulation of ceramides also leads to higher apoptotic effects. Why we, in this study, see a glycosylation of the shorter-chain C6-Cer and C10-Cer, but not of C16-Cer, is an interesting question. Based on the incorporation rates, it is possible that the rate of C16-Cer uptake simply is not fast enough for the glycosylation response to occur. Another possibility that cannot be ruled out is that CholPC may affect how the cells metabolize ceramide. The fact that cholesterol/CholPC vesicles did not have a significant effect on cell viability, even at high concentrations (200 μM), does not exclude the possibility of combinational effects from high levels of ceramide and CholPC. At high concentrations, CholPC may interfere with how the cells process ceramide, inhibiting their glycosylation, and subsequently leading to higher apoptotic effects. This would also explain the lower rates of glycosylation observed in the 200 μM C10-Cer incorporation experiments, when comparing to the corresponding 100 μM C10-Cer experiments.

## Conclusions

The ceramides are a varied molecular species. Distinct enzymes control the chain lengths and saturation of the ceramides, as well as the introduction of functional groups [18, 60–64]. More than 100 distinct mammalian ceramides are detectable using current LC-MS/MS technologies [65]. Because ceramides also exhibit high degrees of compartmentalization in cells, it is plausible that different ceramides may influence different signalling pathways [17–20, 66]. In the present study, we have shown that C6-Cer, C10-Cer and C16-Cer can be introduced into cells by complexation with CholPC. Using this method, we demonstrate that ceramides with different chain lengths clearly exhibit varying rates of cellular uptake. The cellular fate of the externally delivered ceramides, and their subsequent effects on cell viability, may be in part determined by their chain length. Our results suggest that C6-Cer displays an intrinsically stronger anti-proliferative effect when compared to C10-Cer, even when C10-Cer incorporation is equal to, or greater than that of C6-Cer. As the ceramide concentration is increased, the cellular fate of the different ceramides is clearly altered. Both C6-Cer and C10-Cer are glycosylated in HeLa cells at high degrees of cellular uptake. Due to its much slower incorporation rate compared to C6-Cer and C10-Cer, we were unable to determine whether C16-Cer also undergoes such glycosylation. Glycosylation is perhaps a general mechanism by which cells dispose of high amounts of ceramide. A further metabolism of the exogenously added ceramides into GlcCer may obviously also plays a role in the cell viability. While CholPC and cholesterol did not by themselves result in any significant alteration of cell viability, it cannot be excluded that there may be combinational effects between high levels of ceramide and CholPC, affecting cell viability as well as altering how externally delivered ceramide is metabolized. It is also important to note that externally administered ceramides may give rise to significantly different cellular effects, when compared to their endogenously generated counterparts.

## Supporting Information

S1 Fig A representative HPTLC plate of a typical lipid analysis. The HPTLC plate was developed using the solvent system chloroform:methanol:acetone:acetic acid:H_2_O, 10:2:4:2:1 w/w and visualized using orcinol spray and subsequent cupric acid staining. The boxed regions correspond to the different lipids analyzed. The dashed lines represent “Other”, i.e. the traces of radiolabeled lipids between the identified spots.
(TIF)

S2 Fig Inhibition of ceramidase or lysosomal activity. HeLa cells were treated for 45 minutes prior to [^3^H]C16-Cer ([^3^H]palmitic acid labeled) loading (8 hour total ceramide treatment), with ceranib-2 (C2, 20 μM) or chloroquine (CQ, 20 μM). Radiolabel distribution was analysed by HPTLC. The statistical significance compared to the respective controls is indicated with an asterisk (*) p = 0.05.
(TIF)

S3 Fig Inhibition of ceramide synthesis by fumonisin B1. HeLa cells were treated with fumonisin B1 (FB1, 200 μM) for 45 minutes prior to addition of [^3^H]C16-Cer. The ceramide was either **(A)** [^3^H]-labeled in the sphingosine backbone or in the **(B)** palmitic acid portion. Radiolabel distribution was analysed by HPTLC. Total ceramide exposure time was 24 hours.
(TIF)

S4 Fig A representative HPTLC plate of high-dose ceramide treated samples. **(A)** A representative HPTLC plate of the high-dose ceramide treatments (50 μM C6-Cer, 100 μM C10-Cer, 200 μM C16-Cer), illustrating the separation of GlcCer with incorporated chain specific ceramides, from endogenous GSLs. Lane 1, total lipid extract from C6-Cer loaded HeLa cell, lane 2, C10-Cer loaded cells and lane 3, C16-Cer loaded cells. No C16-GlcCer could be detected in total lipid extracts from C16-Cer loaded cells. The plate was developed using the solvent system chloroform:methanol:acetone:acetic acid:H_2_O, 10:2:4:2:1 w/w and stained with orcinol. **(B)** The dot blot was performed to verify that the lipid spots observed in the high-dose C6- and C10-Cer treatments were GlcCer. The HPTLC plate was stained using iodine, and the spots corresponding to C6- and C10-GlcCer were scraped into glass tubes. Lipids were extracted from the silica and absorbed onto a nitrocellulose membrane. Rabbit anti-GlcCer and rabbit anti-GalCer antibodies were used to detect and verify the lipids on the membrane. Commercial standards of GlcCer and GalCer were used as positive and negative controls. The dot blot analysis was repeated twice with identical results.
(TIF)
