# Supplementary figures and images for: Metabolic Conversion of Ceramides in HeLa Cells - A Cholesteryl Phosphocholine Delivery Approach
Source: PLoS One. 2015 Nov 24;10(11):e0143385. doi: 10.1371/journal.pone.0143385 (PMC4658033; doi:10.1371/journal.pone.0143385)

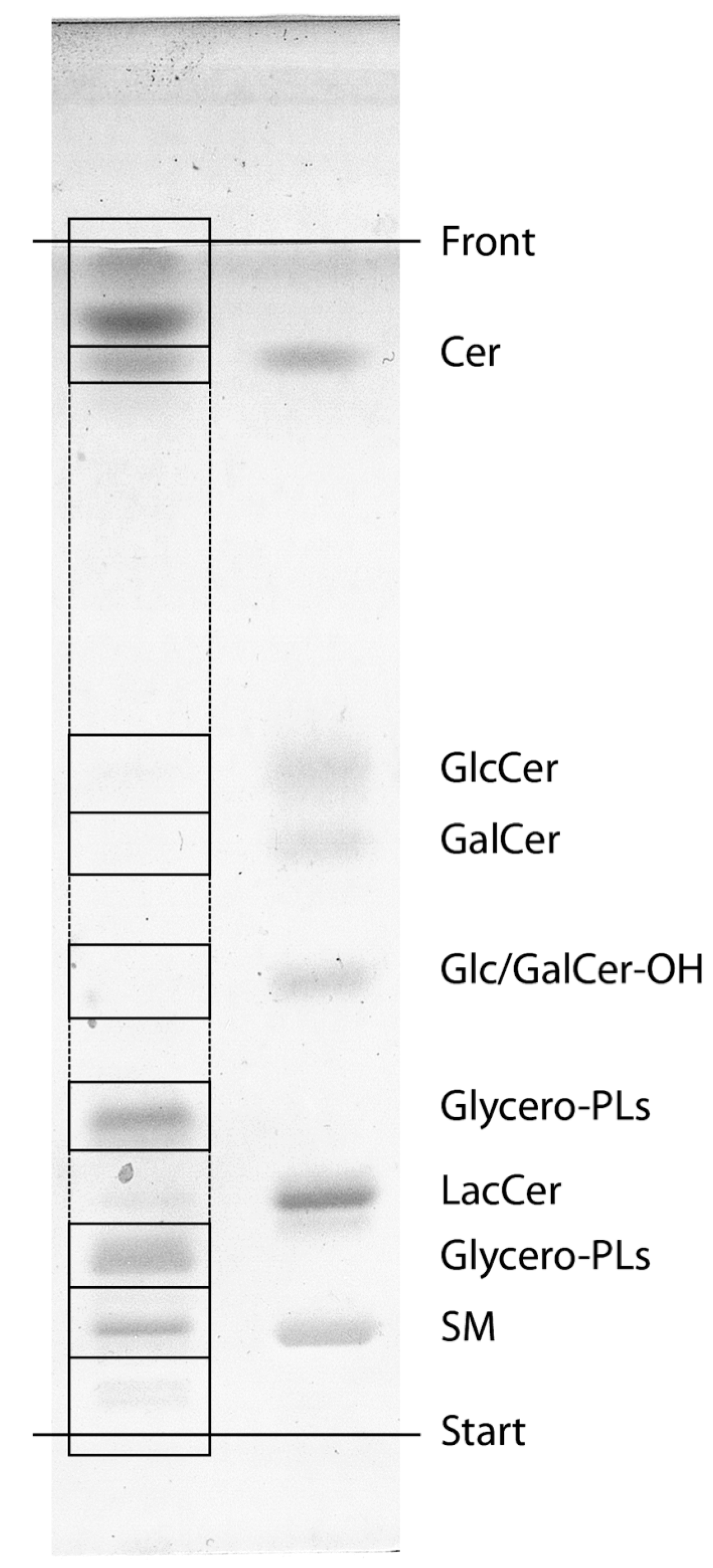

Supplement: S1 Fig — The HPTLC plate was developed using the solvent system chloroform:methanol:acetone:acetic acid:H2O, 10:2:4:2:1 w/w and visualized using orcinol spray and subsequent cupric acid staining. The boxed regions correspond to the different lipids analyzed. The dashed lines represent “Other”, i.e. the traces of radiolabeled lipids between the identified spots. (TIF) [file pone.0143385.s001.tif]

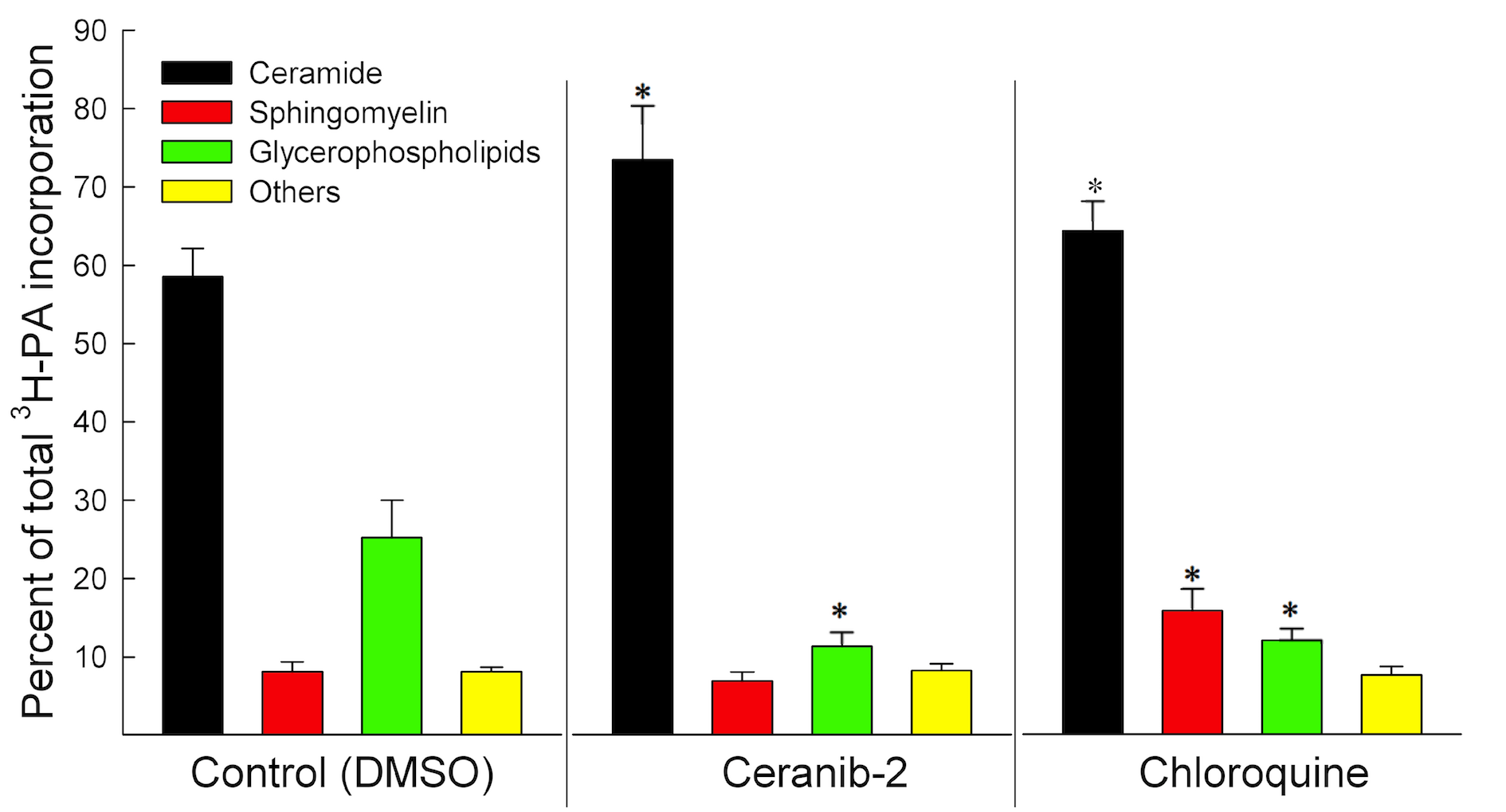

Supplement: S2 Fig — HeLa cells were treated for 45 minutes prior to [3H]C16-Cer ([3H]palmitic acid labeled) loading (8 hour total ceramide treatment), with ceranib-2 (C2, 20 μM) or chloroquine (CQ, 20 μM). Radiolabel distribution was analysed by HPTLC. The statistical significance compared to the respective controls is indicated with an asterisk (*) p = 0.05. (TIF) [file pone.0143385.s002.tif]

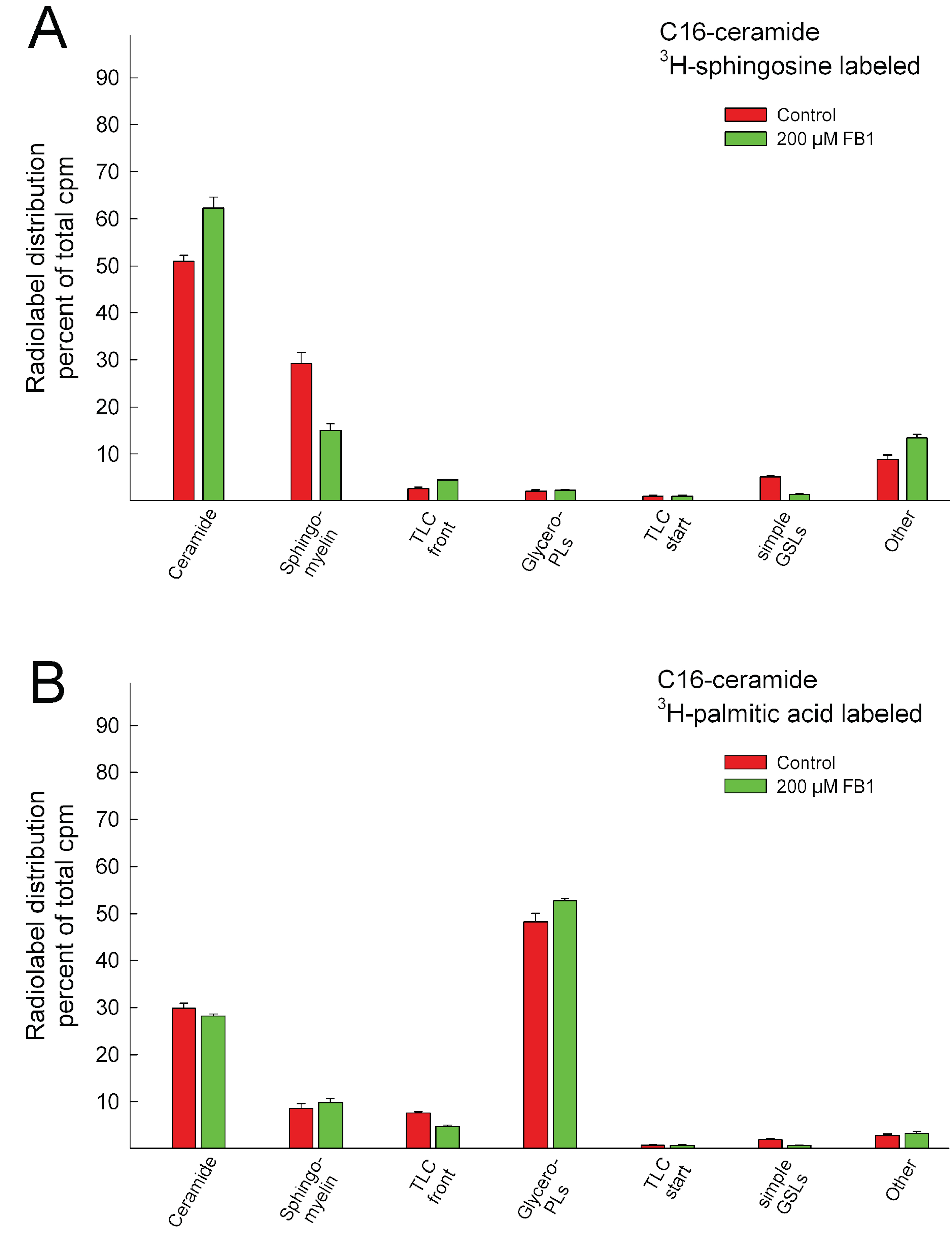

Supplement: S3 Fig — HeLa cells were treated with fumonisin B1 (FB1, 200 μM) for 45 minutes prior to addition of [3H]C16-Cer. The ceramide was either (A) [3H]-labeled in the sphingosine backbone or in the (B) palmitic acid portion. Radiolabel distribution was analysed by HPTLC. Total ceramide exposure time was 24 hours. (TIF) [file pone.0143385.s003.tif]

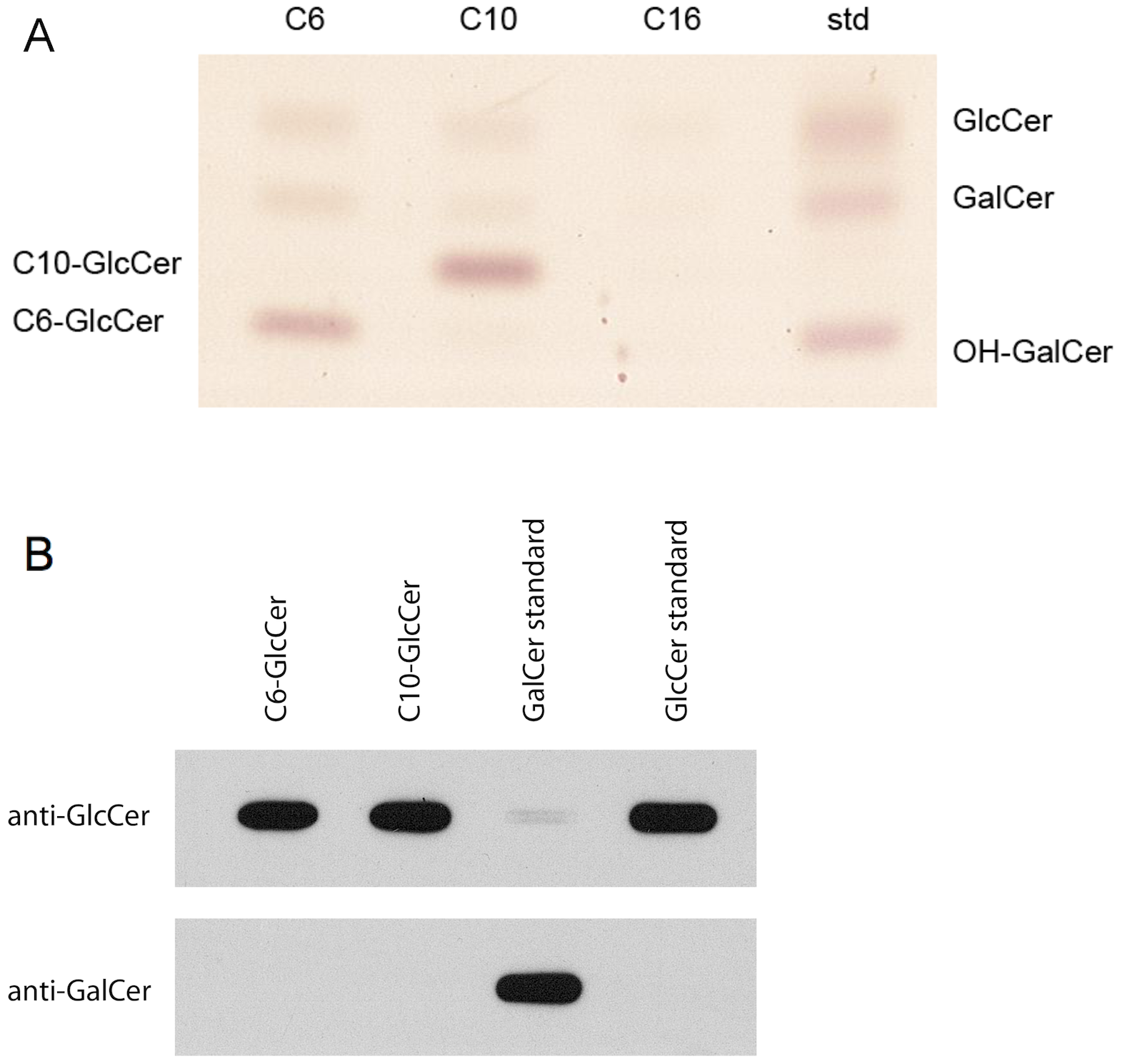

Supplement: S4 Fig — (A) A representative HPTLC plate of the high-dose ceramide treatments (50 μM C6-Cer, 100 μM C10-Cer, 200 μM C16-Cer), illustrating the separation of GlcCer with incorporated chain specific ceramides, from endogenous GSLs. Lane 1, total lipid extract from C6-Cer loaded HeLa cell, lane 2, C10-Cer loaded cells and lane 3, C16-Cer loaded cells. No C16-GlcCer could be detected in total lipid extracts from C16-Cer loaded cells. The plate was developed using the solvent system chloroform:methanol:acetone:acetic acid:H2O, 10:2:4:2:1 w/w and stained with orcinol. (B) The dot blot was performed to verify that the lipid spots observed in the high-dose C6- and C10-Cer treatments were GlcCer. The HPTLC plate was stained using iodine, and the spots corresponding to C6- and C10-GlcCer were scraped into glass tubes. Lipids were extracted from the silica and absorbed onto a nitrocellulose membrane. Rabbit anti-GlcCer and rabbit anti-GalCer antibodies were used to detect and verify the lipids on the membrane. Commercial standards of GlcCer and GalCer were used as positive and negative controls. The dot blot analysis was repeated twice with identical results. (TIF) [file pone.0143385.s004.tif]
